# Exploring patient perspectives on barriers contributing to lost to follow-up in hypertension care: A qualitative study in a tertiary-care hospital in Coastal Karnataka, India

**DOI:** 10.1371/journal.pone.0359571

**Published:** 2026-10-27

**Authors:** Raksha Kamath, Weena Stanley, Partha Protim Hazarika, Shwetha Tumkur Shivakumar, Bhageerathy Reshmi

**Affiliations:** 1 Department of Health Information Management, Manipal College of Health Professions, Manipal Academy of Higher Education, Manipal, India; 2 Department of Medicine, Kasturba Medical College, Manipal Academy of Higher Education, Manipal, India; 3 Department of Health Informatics, MGM School of Biomedical Sciences, MGM Institute of Health Sciences (MGMIHS), Navi Mumbai, India; 4 Department of Psychology, Manipal College of Health Professions, Manipal Academy of Higher Education, Manipal, India; Kasturba Medical College Manipal, INDIA

## Abstract

**Background:**

Hypertension requires lifelong treatment and regular follow-up to prevent complications; however, lost to follow-up (LTFU) remains a major challenge in long-term hypertension care, particularly in resource-constrained settings.

**Objective:**

This study explored patient perspectives on barriers contributing to LTFU in hypertension management.

**Methods:**

A qualitative study was conducted among 32 hypertensive patients who were LTFU at a tertiary care hospital in Coastal Karnataka, India. Participants were aged 31–69years, with 21 (65.6%) men and 11 (34.4%) women. Participants were identified through hospital records and interviewed using a semi-structured interview guide. Interviews were conducted from February to April 2026 Interviews were audio-recorded, transcribed verbatim, and analysed thematically following COREQ guidelines.

**Results:**

Six major themes emerged.: (1) illness perception and self-management practices, (2) care-seeking and follow-up behaviour, (3) structural and socioeconomic barriers, (4) health system and continuity gaps, (5) patient-perceived strategies to improve continuity of care, and (6) health information continuity and interoperability needs. Participants described symptom-driven healthcare seeking, medication non-adherence, occupational responsibilities, and competing family priorities contributing to irregular follow-up. Financial burden, including treatment costs, transportation expenses, and wage loss, substantially affected continuity of care. Long travel distances, dependence on family members, overcrowding, rushed consultations, and inadequate follow-up mechanisms further influenced disengagement from care. Participants also reported fragmented healthcare utilisation, repeated investigations, dependence on paper-based records, and poor continuity of medical information across healthcare facilities. Several participants perceived that integrated digital health information systems, decentralised services, appointment reminders, and improved counselling could strengthen long-term hypertension follow-up.

**Conclusions:**

LTFU in hypertension care is influenced by interconnected individual, socioeconomic, and health system-related barriers. A notable finding was the fragmented nature of continuity of care and poor health information continuity across healthcare facilities. Strengthening patient-centred follow-up systems, decentralised chronic disease services, financial protection mechanisms, and interoperable health information systems may improve continuity of hypertension care and reduce long-term disengagement from chronic disease management.

## 1. Introduction

Hypertension is a major risk factor for cardiovascular disease, contributing to approximately 10.8 million deaths globally each year [1,2]. It significantly burdens health systems, particularly in low- and middle-income countries (LMICs), where control rates remain suboptimal despite available treatments [3]. In India, hypertension prevalence has increased with lifestyle changes and urbanisation [4]), affecting nearly one in four individuals according to NFHS-5, with many remaining untreated or inadequately treated [5]. Despite national programmes, gaps in long-term follow-up and treatment adherence persist [6].

Lost to follow-up (LTFU), referring to patients who do not return for scheduled follow-up care within a defined period, is a critical but underexplored issue in hypertension management [7]. LTFU disrupts continuity of care, leading to poor blood pressure control and increased risk of complications [8]. Studies from LMICs report substantial missed follow-up among patients with hypertension, highlighting challenges in retaining patients in care [9]. However, the timeframe used to define LTFU varies across studies and healthcare settings, and there is no single universally accepted duration for defining LTFU in hypertension care. In the present study, LTFU was defined as no further follow-up or contact with the hospital for ≥12 months from the next follow-up visit advised by the treating physician. This definition was based on the available literature and was also supported by the views of healthcare professionals regarding the follow-up practices in the study setting.

LTFU in hypertension is multifactorial, involving socio-demographic, economic, cultural, and health system factors [10]. Financial constraints, including out-of-pocket expenses (OOPE) for consultations, medications, and transport, significantly limit care in resource-poor settings [11]. Low health literacy, the asymptomatic nature of hypertension, and cultural beliefs contribute to poor risk perception and reduced adherence [12,13]. Health system challenges such as fragmented care, long waiting times, and poor provider communication further reduce engagement with services [14,15]. These barriers are particularly evident in rural and underserved populations with limited access to continuous care [16]. Continuity of care is a key component of Universal Health Coverage (UHC), ensuring equitable access to quality services without financial hardship.

While quantitative studies highlight poor adherence and follow-up, they often fail to capture patients’ lived experiences of managing hypertension [17]. Understanding patient perspectives—including beliefs, challenges, coping strategies, and unmet needs—is essential for designing patient-centred interventions, best explored through qualitative methods [18]. Such studies remain limited in the Indian context, particularly in real-world settings, highlighting the need to explore disengagement from care. Insights into patient-perceived barriers can guide the development of culturally appropriate and sustainable interventions to improve adherence and follow-up [19].

Therefore, this study aims to explore patient perspectives on barriers contributing to LTFU in hypertension care, focusing on experiences, challenges, and unmet needs. The findings aim to inform patient-centred, context-specific interventions to improve continuity of hypertension care.

## 2. Materials and methods

### 2.1. Study setting

This qualitative study was carried out at, a tertiary care teaching hospital in coastal Karnataka, serving a socio-demographically diverse population functioning as a key referral centre for urban and rural areas. The hospital receives patients referred from primary and secondary healthcare facilities for specialist evaluation and further management.

Hypertension management in the Indian public health system is supported by national programmes and treatment guidance, including the National Programme for Prevention and Control of Non-Communicable Diseases (NP-NCD) and the Government of India’s standard treatment guidance for hypertension. At the study site, hypertension management is guided by the 2025 American Heart Association/American College of Cardiology (AHA/ACC) Guideline for the Prevention, Detection, Evaluation, and Management of High Blood Pressure in Adults.

The study was conducted between February to April 2026 following ethical approval from the Institutional Ethics Committee of Kasturba Hospital (IEC1:263/2024), Manipal, and the study was registered prospectively under the clinical trial Registry of India CTRI/2024/09/073775. All participants gave verbal informed consent via telephone after being informed of the study objectives and their rights, as stated in the participant information sheet.

### 2.2. Study design and data collection

A qualitative design using purposive sampling explored patient perceptions of barriers leading to LTFU in hypertension care. Qualitative approaches are appropriate for understanding experiences and contextual barriers in chronic disease care [20].

Adult patients diagnosed with hypertension who had previously taken treatment at this tertiary care hospital but not returned for follow-up or had no further contact with the hospital for ≥12 months from the next follow-up visit advised by the treating physician were identified from hospital records and included in the study. This 12-month period was used as the operational definition of LTFU in the present study, based on the available literature and follow-up practices in the study setting. As they had not attended scheduled follow-up visits within this 12-month period, they were considered “lost to follow-up” [21].

Access to hospital records was obtained following institutional permissions and eligible participants were identified, and their contact details retrieved. The first author, a female research scholar, contacted the eligible participants by telephone and invited them to participate in the study. A total of thirty-nine individuals were approached for participation of these, two refused participation and five were deceased, and the remaining thirty-two were interviewed. Purposive sampling was used to identify participants who met the predefined LTFU criteria and were willing to participate. Participants were selected to capture variation in age and patterns of disengagement from care, including those who had recently become LTFU, those who had been out of care for several years, and those who had returned to care after a prolonged gap but subsequently missed follow-up again. Participants with varying durations since hypertension diagnosis and treatment were included. There was no prior relationship between the researcher and the participants before recruitment. Before the interview, participants were informed that the interviewer was a research scholar conducting the study to explore barriers contributing to discontinuity in hypertension care.

In-depth interviews (IDI) were conducted by the first author, a research scholar involved in the study of hypertension care continuity. The researcher had prior understanding of hypertension care and continuity of care, which informed the research process. To support reflexivity, the researcher maintained reflexive notes throughout the research process. The researcher was not involved in the clinical care of the participants A semi-structured interview guide was used consistently. The guide was developed based on a literature review and expert recommendations from public health and clinical professionals. It explored the following areas: reasons for discontinuing follow-ups, barriers to accessing healthcare, knowledge of managing hypertension, perceptions of disease severity and treatment, and patient needs and expectations.

The guide was reviewed for content validity, clarity, and relevance, and refined accordingly and translated into the local language Kannada by bilingual translators to facilitate effective communication.

Following expert validation, the patient IDI guide was pilot tested with six individuals with hypertension who had experienced disruption in follow-up care. The pilot interviews were conducted telephonically to check the clarity, understanding, order of the questions, and interview duration. Minor changes were made to the order of questions based on the feedback received. Data from the pilot interviews were not included in the final analysis. The semi-structured interview guide is provided in; S1 File. Semi-structured patient interview guide.

The first author conducted all interviews telephonically at participants’ convenience, with each participant interviewed once. Participants’ willingness to participate and consent for audio recording were obtained before each interview. When participants did not consent to audio recording, the interview was not recorded and reflexive notes were taken. Interviews lasted 15–30 minutes and were conducted in Kannada. As the interviews were conducted telephonically, the lack of face-to-face interaction may have limited the depth of some responses, particularly when participants had limited time or were engaged in work or other activities. Follow-up questions were used to encourage participants to elaborate and clarify their responses where needed. The interviews were transcribed verbatim in Kannada and translated into English. The English translations were reviewed during the analysis process to ensure that the meaning of participants’ responses was retained. Quotations were translated into English while preserving the original meaning. Saturation was assessed during ongoing data collection and analysis. Thematic saturation was considered reached after the 28th interview, when no new themes emerged [22]; four additional interviews were conducted to confirm coding stability. The study adhered to COREQ guidelines to ensure methodological rigor and transparency [23]: **S2 File**. COREQ checklist.

### 2.3. Data Analysis

Data were analysed using thematic analysis as described by Braun and Clarke [24]. Transcripts were read repeatedly for familiarisation, followed by systematic coding, theme development, review, and refinement.

An inductive approach was used, allowing themes to emerge directly from data without predefined frameworks [25]. Coding involved familiarisation, generation of initial codes, grouping into categories, and development of themes and subthemes.

Intercoder reliability was ensured through independent coding of 30% of transcripts by two researchers (RK and BR) to improve accuracy. Differences were resolved through discussion, achieving 90% agreement. A coding framework and codebook were developed iteratively, and a framework matrix was created using Microsoft Excel (version 21). Atlas ti version 9 supported data organisation and analysis. Rigour was ensured through intercoder verification, peer debriefing, documentation of coding decisions, and regular discussions to ensure consistency and credibility of findings. Themes were refined to align with patient experiences of barriers leading to LTFU in hypertension care, with emphasis on identifying system-level gaps relevant to continuity of care. Participant quotations were anonymised using age and gender identifiers to maintain confidentiality.

## 3. Results

A total of 32 participants previously diagnosed with hypertension and LTFU from care at the tertiary care hospital participated in the study. Table 1 depicts the demographic characteristics. The participants represented diverse socioeconomic and occupational backgrounds, including daily wage workers, the elderly, homemakers, and employed individuals from semi-urban and rural areas. Analysis showed that disengagement from hypertension care was influenced by interconnected individual, social, and health system-related factors. Analysis identified six major themes and their corresponding subthemes, as shown in Table 2.

**Table 1:** Sociodemographic and clinical characteristics of hypertensive patients who were lost to follow-up (n = 32).

Characteristics	Frequency (n = 32)	Percentage (%)
**Age Group (years)**		
<40	5	**16%**
40–49	9	**28%**
50–59	11	**34%**
≥60	7	**22%**
**Sex**		
Male	21	**66%**
Female	11	**34%**
**Place of Residence**		
Rural	15	**47%**
Urban/Semi-urban	17	**53%**
**Occupation**		
Daily wage worker	16	**50%**
Homemaker	9	**28%**
Employed/Salaried	6	**19%**
Retired	2	**6%**
**Duration of Hypertension**		
<5 years	4	**13%**
5–10 years	9	**28%**
>10 years	19	**59%**
**Follow-Up Status**		
Gap in care ^a^	1	**3%**
Completely discontinued follow-up from this tertiary care setting ^b^	31	**97%**
Current Source of Care*		
Local clinic/private practitioner	30	**94%**
Government facility	29	**91%**
Multiple providers	19	**59%**
No regular care	3	**9%**
Presence of Comorbidities*		
Diabetes mellitus	30	**94%**
Cardiovascular disease ^c^	26	**81%**
Kidney-related complications	29	**91%**

**Note::** * *Multiple responses were recorded for selected characteristics;* ^*a*^*“Gap in care” refers to a participant who had previously experienced a prolonged interruption in follow-up, subsequently returned to the tertiary care hospital, and later became LTFU again;* ^*b*^*“Completely discontinued follow-up from this tertiary care setting” refers to participants who had no further follow-up or contact with the hospital for ≥12 months from the next follow-up visit advised by the treating physician;* ^*c*^*Cardiovascular disease and kidney-related complications were classified based on documented heart-related and kidney-related conditions, respectively.*

**Table 2:** Overview of themes and subthemes.

Themes	Subthemes
**Theme 1:** Illness Perception and Self-Management Practices	Knowledge and Risk Perception
	Treatment Behaviour and Self-Management
	Social and Interpersonal Influences
**Theme 2:** Care-Seeking and Follow-Up Behaviour	Disrupted Follow-Up Patterns
	Care Navigation and Healthcare Utilisation
**Theme 3**: Structural and Socioeconomic Barriers	Financial and Occupational Constraints
	Geographic and Access Barriers
**Theme 4:** Health System and Continuity Gaps	Service Delivery Constraints
	Administrative and Follow-Up Gaps
	Fragmented Information and Care Continuity
Theme 5: Patient-Perceived Strategies to Improve Continuity of Care	Administrative Strengthening
	Financial Protection Improvements
	Communication and Decentralised Care
Theme 6: Health Information Continuity and Interoperability Needs	Fragmented Medical Records Across Facilities
	Repetition of Investigations and Duplication of Care
	Dependence on Paper-Based Records
	Need for Integrated Digital Health Information Systems
	Lack of Coordination Between Levels of Care

**Theme 1: Illness Perception and Self-Management Practices**.

Participants demonstrated varying understanding of hypertension, its complications, and the importance of regular follow-up. Many perceived hypertension as serious only when symptoms were present, resulting in irregular treatment and follow-up.

Symptom-based understanding of hypertension was common, with treatment often linked to symptoms such as headache or dizziness. Some participants viewed hypertension as an age-related condition rather than a chronic disease requiring continuous monitoring. Although some participants were aware of complications, long-term self-management remained challenging due to competing priorities.

*“When there are no symptoms, I feel it is okay… so I postpone going.” (Patient,48y,female)*

*“BP is common at this age… everyone has it.” (Patient,69y,male)*

*“I know I should take tablets regularly… but sometimes because of work I forget.” (Patient,35y,female)*

Medication non-adherence was associated with forgetfulness, work responsibilities, irregular routines, and absence of symptoms. Dietary modification and exercise were difficult to sustain due to household and occupational responsibilities, although some reported practising walking, yoga, and dietary control. Financial and work-related priorities often delayed care seeking particularly among daily wage earners. Some participants also reported use of alternative treatments during discontinuation.

*“If BP becomes normal, I stop tablets for some days.” (Patient,69y,male)*

*“I was going to an Ayurvedic doctor… so I didn’t restart allopathic treatment.” (Patient,54y,male)*

Family support facilitated healthcare access through transport and reminders, while household responsibilities limited self-care. Advice from relatives and community members influenced treatment decisions and provider switching.

*“If someone is there to bring me, then I go.” (Patient,83y,male)*

*“There is nobody else to take care of the family work.” (Patient,45y,female)*

**Theme 2: Care-Seeking and Follow-Up Behaviour**.

Participants reported irregular and fragmented healthcare use driven by work, finances, and low perceived need, and symptom-driven care-seeking. Routine follow-up was interrupted by work commitments, travel burden, financial constraints, and low perceived need for care and sought care only when symptoms worsened.

*“I planned to come, but because of work it kept getting postponed.” (Patient,62y,male)*

*“I went when I felt uneasy.” (Patient,47y,male)*

Few gradually discontinued tertiary care visits after missed appointments.

*“One visit got missed, then slowly I stopped going regularly… but I follow at my place.” (Patient,55y,female)*

Participants frequently switched providers based on accessibility, affordability, and convenience, often preferring local and government facilities, while elderly participants depended on family members to facilitate healthcare visits.

*“Later, I started going to a nearby hospital.” (Patient,23y,male)*

*“If my son is free, then only I can come.” (Patient,83y,male)*

**Theme 3: Structural and Socioeconomic Barriers**.

Financial and geographic barriers were major contributors to LTFU.

Participants described substantial OOPE related to consultations, medicines, investigations, travel, and food expenses during hospital visits. Daily wage workers were particularly affected to balance healthcare needs with occupational responsibilities. Travel-related expenses further affected continuity of care.

*“Around ₹2,000–3,000 per month. If I get tests done, it costs more.” (Patient,48y,female)*

*“If I take leave, there is no salary.” (Patient,35y,female)*

Travel distance and time, poor transport connectivity, physical exhaustion, multiple transport changes further reduced follow-up, especially in rural and elderly individuals. Transportation challenges were particularly common among elderly individuals and those with mobility limitations.

*“By the time we reach the hospital, half the energy is gone.” (Patient,48y,male)*

**Theme 4: Health System and Continuity Gaps**.

Long waiting times, overcrowding, rushed consultations, inadequate explanations and perceived disrespectful communication from healthcare personnel were reported to reduce motivation and follow-up adherence.

*“By the time we meet the doctor, the whole day is over.” (Patient,44y,female)*

*“Nobody explained properly why regular follow-up is important. They were not polite when speaking to us.…” (Patient,52y,male)*

Missed appointments were rarely followed by reminders, and participants reported uncertainty regarding referral pathways and follow-up schedules, which contributed to frustration and disengagement from care.

*“I never know exactly what to do… and it becomes frustrating.” (Patient,58y,male)*

Participants reported repeated histories, paper-based records, and repeated investigations due to lack of record integration which led to fragmented care and poor continuity of health information across multiple healthcare facilities. These experiences contributed to fragmented care and increased the burden of navigating long-term hypertension management.

*“Every hospital asks for different reports, and we have to explain the same history again and again.” (Patient,39y,male)*

*“We carry one big file everywhere… sometimes papers get lost.” (Patient,35y,female)*

Some perceived digital records as beneficial to strengthen continuity of care, though awareness remained limited.

*“If doctors can see everything online, there would be no need to carry reports again.” (Patient,47y,male)*

Fragmented care continuity increased patient burden and disrupted long-term follow-up.

**Theme 5: Patient-Perceived Strategies to Improve Continuity of Care**.

Participants suggested several strategies to strengthen continuity of hypertension care, including improved appointment scheduling and follow-up tracking, financial protection, better communication, and decentralised care.

Appointment reminders, structured scheduling systems, and follow-up tracking mechanisms were frequently recommended.

*“If there is a reminder, people may not forget.” (Patient,39y,male)*

The need for lower OOPE, simplified insurance procedures, and subsidised investigations and medications were highlighted.

*“Government hospital is cheap madam… so I go there. Here private feels costly.” (Patient,28y,male)*

Participants emphasised; counselling on the chronic nature of hypertension, decentralised follow-up services, and better coordination between local and tertiary healthcare facilities. They felt that clear explanations could improve understanding and engagement, while access to services closer to home could reduce the burden of travelling to tertiary facilities.

*“If someone explains clearly, patients may take it more seriously.” (Patient,28y,male)*

*“It would be helpful if check-ups are available nearer to home.” (Patient,69y,male)*

**Theme 6: Health Information Continuity and Interoperability Needs**.

Participants reported difficulty maintaining continuity of medical information across multiple healthcare providers. Lack of coordinated record sharing led to repeated medical histories and fragmented follow-up care.

*“Every hospital asks for old files again… sometimes we cannot find them.” (Patient,49y, female)*

Lack of shared medical records resulted in repeated investigations and duplication of care, increasing financial burden and inconvenience. Repeated investigations created frustration and disrupted continuity of care among participants.

*“Even after doing tests before, they asked to repeat everything.” (Patient,55y, female)*

Participants relied on physical medical records, which were often misplaced or unavailable during consultations. This reliance placed responsibility on patients to maintain and transport records across healthcare facilities.

*“We carry one big file everywhere… sometimes papers get lost.” (Patient,35y, female)*

Some participants perceived digitally accessible medical records as useful for improving continuity of care and reducing repeated investigations. Integrated digital systems were perceived to improve navigation and continuity of care.

*“If doctors can see everything online, it will become easier. There would be no need to carry reports again and again.” (Patient,47y, male)*

Participants reported poor coordination between healthcare facilities, resulting in fragmented care and inconsistent follow-up.

*“After going back to the nearby hospital, there was no connection with the treatment here.” (Patient,54y, male)*

## 4. Discussion

This qualitative study explored the complex interaction of individual, socioeconomic, and health system-related factors influencing continuity of hypertension treatment and patient perceptions for disengagement from follow-up care. The findings suggest that LTFU results from multiple interconnected barriers, including poor illness perception, competing livelihood priorities, fragmented healthcare experiences, financial hardship, and gaps in long-term chronic disease management systems.

Participants commonly perceived hypertension through symptoms, believing treatment and follow-up were necessary only during episodes of headache, weakness, or uncontrolled blood pressure which that interfered with their day-to-day activities. Consequently, medications and follow-up visits were often discontinued once symptoms subsided or blood pressure appeared controlled. Similar findings have been reported in previous studies, where poor adherence was linked to low perceived severity and the asymptomatic nature of hypertension [26,27], highlighting ongoing gaps in patient knowledge regarding its long-term effects and silent progression. Participants also described difficulties sustaining long-term self-management practices, including medication adherence, dietary modification, exercise, and stress management, because of forgetfulness, work-related commitments, and competing priorities. Comparable challenges have been observed in LMIC settings, particularly among individuals engaged in informal employment [27,28]. In addition, our study participants perceived lifestyle modifications such as yoga and dietary control as substitutes for antihypertensive medication, contributing to self-discontinuation of treatment. Inadequate counselling regarding the need for lifelong treatment further contributed to inconsistent adherence practices.

Socioeconomic barriers including transportation costs, consultation fees, lost wages, and travel burden significantly affected follow-up continuity. Daily wage workers frequently prioritised work over healthcare visits, while rural participants described substantial travel-related difficulties. Similar barriers have been reported in other resource-constrained settings, including Ghana [29]. Participants also demonstrated fragmented healthcare utilisation, frequently switching between providers based on accessibility, affordability, and symptom severity. Such fragmented care-seeking behaviour is known to adversely affect treatment continuity and long-term blood pressure control [30]. Many of our study participants additionally relied on local facilities or self-management and sought tertiary care primarily during acute illness requiring emergency care, increasing both patient and health system burden.

Health system-related barriers including long waiting times, overcrowding, rushed consultations, and poor patient–provider communication negatively influenced follow-up continuity, consistent with previous studies [26,27]. These factors, together with inadequate counselling regarding the need for lifelong follow-up, further contributed to disengagement from care. Unlike earlier studies focusing mainly on individual-level adherence barriers, the present study additionally highlights fragmentation in continuity of care and health information flow across healthcare facilities as important contributors to LTFU. Participants frequently described carrying paper-based records, repeating investigations, and repeatedly explaining their medical history across multiple providers. Although some participants perceived digitally accessible records as beneficial for continuity of care, awareness regarding such systems remained limited among several individuals [28,29].

Participants suggested several strategies to improve continuity of hypertension care, including appointment reminders, decentralised follow-up services, improved counselling, simplified insurance procedures, and integrated digital medical records. These findings support growing evidence favouring patient-centred chronic disease care models that strengthen long-term adherence through community-level follow-up, digital reminder systems, and improved healthcare coordination [8,31]. Integrated and interoperable health information systems may further strengthen continuity of care by reducing fragmented care, duplication of investigations, and patient burden.

The findings have important implications for strengthening hypertension care delivery in resource-limited settings. Individual-level adherence counselling alone may be insufficient to address LTFU. Broader strategies including patient education, decentralised services, structured follow-up mechanisms, enhanced provider communication, financial protection measures, and interoperable health information exchange systems are also necessary to support longitudinal chronic disease management and continuity of care across healthcare settings by enabling secure sharing of patient information. These measures may additionally contribute towards strengthening coordinated chronic disease services and broader UHC goals.

This study has certain limitations. As it was conducted in a single tertiary care setting, the findings may not be transferable to all hypertensive populations or healthcare settings. Experiences of individuals who could not be reached or declined participation may differ. Recall bias and subjective interpretation may also have influenced self-reported narratives. Nevertheless, the study provides important contextual insights into barriers and lived experiences associated with LTFU in hypertension care and may help inform patient-centred strategies to strengthen continuity of hypertension management.

## 5. Conclusion

This study highlights how interconnected individual, social, and health system constraints contribute to LTFU in hypertension care and disrupt continuity of treatment. The findings particularly highlight fragmentation of care and health information flow across healthcare facilities as important contributors to long-term disengagement from hypertension management.

Strengthening patient-centred chronic disease care through improved counselling, decentralised follow-up services, structured follow-up mechanisms, financial protection measures, enhanced provider communication, and interoperable health information systems may help reduce fragmentation and improve follow-up retention. Maintaining continuity of care across healthcare settings is important for improving long-term hypertension management and supporting broader UHC goals.

## Supporting information

S1 File Semi-structured patient interview guide. (DOCX)

S2 File COREQ checklist. (PDF)

## Abbreviations

LTFU: Lost to follow up
UHC: Universal health coverage
LMIC’s: Low- and middle-income countries
IDI: In- depth interviews
COREQ: Consolidated Criteria for Reporting Qualitative Research
OOPE: Out of pocket expenditure
