# Supplementary material for: Exploring patient perspectives on barriers contributing to lost to follow-up in hypertension care: A qualitative study in a tertiary-care hospital in Coastal Karnataka, India
Source: PLoS One. 2026 Oct 27;21(10):e0359571. doi: 10.1371/journal.pone.0359571 (PMC13712698; doi:10.1371/journal.pone.0359571)
Supplement: Supplementary file 2 [file pone.0359571.s001.docx]

**“Barriers to Continuity of Hypertension Care: In-Depth Interview Guide for Lost-To-Follow-Up Patients (BCHC-IDIG)”**

**Introduction:**

Dear Participant,

Thank you for joining the conversation and participating in this study.

I am conducting a study to understand why people with hypertension and its associated co- morbid conditions be lost to follow up for care, and to know what are the barriers, challenges and reasons in missing the appointments.

This is to request you to participate since your point of view as a person with hypertension is important to understand and explore the reasons and barriers behind being lost to follow-up for care. I sincerely appreciate your consent for participating in this discussion.

“Our conversation may last about 20 to 30 minutes.”

Your answers will be kept confidential. I will record this session so I can listen carefully instead of taking notes. The recording will only be used for this study and will be deleted as per guidelines. There are no right or wrong answers. It is your perspective and we respect your opinion. If you agree to this interview and recording, please sign this consent form. We will start this interview only after your consent.

“Shall we begin the interview?”

**The purpose of the interview:**

This study aims to understand why patients stop coming for follow-up care and how this affects the quality of healthcare and patient health. We want to know from patients with high blood pressure about the problems they face in following and sticking to their treatment as scheduled.

**Participant information sheet**

**Consent form**

**Questions**

**INTERVIEW QUESTIONS:**

1) How much do you know about your illness, and how to take care of it?

Where did you get first diagnosed with your illness

How did you go forward with the treatment once you got diagnosed with it

Awareness about the disease and it co morbid conditions (appropriate education & awareness)

How often do you get your health checked

Awareness regarding that, this condition requires long term treatment

2) How much do you know about the importance of regular checkup visits (follow up care)?

Probes:

Information regarding the follow up process

Awareness about the follow up and disease condition (necessary information, appropriate education, awareness)

About the investigations and procedure done and what are the benefits performing it & how it will be useful in diagnosis and treatment of the patient’s disease condition

3) Why did you miss or stop coming to your check-up visits? How many times do you think that you did not follow up as per the schedule?

Difficulties that are faced in sticking to follow up appointments (investigations, procedures, treatment as suggested by doctors)

Reasons for not coming and sticking to check up visits (reasons for being lost to follow up)

**Probes:**

Financial burden/cost of drugs and healthcare

Relocation

Insurance - Uninsured or reimbursement, which mode

Side effects of drugs – worsen disease state

Household responsibilities (Caregiving for any family member)

Lack of awareness

Level of education

Age

Fear

Travel cost

Mental health factors

Perception about disease

Social stigma

Denial

Distance

Schedule and time flexibility

Stress

Complexity of hospital and changes in set up

Lack of caregiver or physicians

4) Since your last visit to the hospital or clinic, can you explain how you take your medicines every day?

Probes:

Taking medications as prescribed on the last visit

Complete withdrawal from medications

Relying on other forms of medications

5) Which hospitals or clinics have you visited since you found out you have high blood pressure? Probes:

Primary care

Secondary care

Tertiary care settings

6) How have you been utilizing these healthcare facilities for managing your health condition?

Probes:

Regarding health insurance

Hospital visits (primary, secondary, tertiary)

Number of times they go for checkups

Increased number of hospitalizations

Increased re- admission rate

Increased emergency room visits or use

Has your health insurance affected the way you use hospital or emergency services?

7) While you missed check-ups and were lost to follow up at this hospital, were you taking treatment or medicine from any other doctors or did you try any other treatments? Were you sent to another specialist for care?

Probes:

Contacting primary care physicians (self)

Referral to other specialty physician’s

Taking allopathy and homeopathy medications or any other indigenous treatment modalities

8) Can you talk about the money you spend on your treatment, like medicine, hospital visits, travel, or other healthcare costs?

Total medication costs (monthly expenses)

Travel costs per visits

Other costs like (doctors consultation fees (second opinions), medical laboratory test, medical equipment’s, insurance costs, home care costs (home nurse/ bystanders), alternative treatment costs

Missed work or income costs

9) How is your family supporting you in managing your health condition and for attending follow-up visits?

Probes:

Sole earner, financial and mental support

Support and encouragement for follow up visits

10) What initiatives and factors would help you keep going to your check-ups and stay on treatment at the same hospital?

**Thank you!**
